# Trends in *Enterococcus faecium* Bacteremia: Exploring Risk Factors with Emphasis on Prior Antibiotic Exposure

**DOI:** 10.3390/microorganisms12101932

**Published:** 2024-09-24

**Authors:** Erik Sörstedt, Gustaf Ahlbeck, Ulrika Snygg-Martin

**Affiliations:** 1 Department of Infectious Diseases, Institute of Biomedicine, Sahlgrenska Academy, University of Gothenburg, 405 30 Gothenburg, Sweden; erik.sorstedt@vgregion.se (E.S.);; 2 Region Västra Götaland, Department of Infectious Diseases, Sahlgrenska University Hospital, 416 50 Gothenburg, Sweden; 3 Centre for Clinical Research, Västmanland Hospital Västeras, 721 89 Västerås, Sweden; 4 Centre for Antibiotic Resistance Research (CARe), University of Gothenburg, 405 30 Gothenburg, Sweden

**Keywords:** enterococcal bacteraemia, antibiotic resistance, *Enterococcus faecium*, piperacillin/tazobactam

## Abstract

Enterococcal bacteremia (EB) is on the rise both in Sweden and globally. While *Enterococcus faecalis* (*E. faecalis*) is susceptible to ampicillin and piperacillin/tazobactam (pip/taz), *Enterococcus faecium* (*E. faecium*) is not. Historically, most enterococcal infections have been caused by E. faecalis, but the epidemiology is changing with increasing recognition of enterococci as nosocomial pathogens and the emergence of resistance to commonly used antimicrobial agents. The use of pip/taz has increased dramatically in Sweden, but it is unknown if this has affected the relative incidence of *E. faecalis/E. faecium* bacteremia. Here, we investigate whether the number and proportion of *E. faecium* bacteremia (EfmB) cases have increased. Additionally, risk factors associated with EfmB with a focus on prior antibiotic exposure are analyzed. Medical journals of 360 patients with EB admitted to Sahlgrenska University Hospital are reviewed. The proportion of EfmB cases increased from 41% in 2015 to 51% in 2021. Hospital-acquired infection, previous exposure to pip/taz, and carbapenems are identified as independent risk factors for EfmB. There are considerable patient-related differences between the EfmB and EfsB groups, but there is no difference in mortality rates. In conclusion, the increasing proportion of EfmB cases is concerning and is seen parallel to the expanding use of pip/taz, one possible contributing factor. Our findings suggest that a cautious approach to antibiotic use is essential to prevent the spread of antibiotic-resistant bacteria.

## 1. Introduction

Enterococci are Gram-positive, non-spore-forming, facultative anaerobic bacteria that inhabit the gastrointestinal tract of humans and other animals. Enterococci also have the ability to persist in the environment and resist disinfection procedures, resulting in a widespread distribution in clinical settings [1]. Historically, enterococci have belonged to the *Streptococcus* group D, but in 1984, Schleifer and Kilpper-Bälz proposed the new genus *Enterococcus*, which should include the former *Streptococcus faecalis* and *Streptococcus faecium* [2]. This was based on differences in nucleic acid homology and in antibiotic-resistance patterns. Enterococci share several characteristics with streptococci, but a number of key reactions, used for biochemical identification but also implicating biological differences, are typical for the genus *Enterococcus* [3].

Although more than 50 different species of enterococci have been described, the predominant species causing human infections are *E. faecalis* and *E. faecium*, with the latter generally being a less prevalent cause of bloodstream infections, although proportions vary largely by geographical region and over time [1]. Hence, historically, the majority of invasive enterococcal infections have been caused by *E. faecalis*, but in recent decades, the epidemiology of enterococcal bacteremia (EB) has been identified, such as the recognition of enterococci as increasingly important nosocomial pathogens and, in addition to their intrinsic resistance to many antibiotics, the emergence of additional acquired resistance mechanisms to commonly used antimicrobial agents [4]. Hospital-acquired enterococcal bacteremia has also been shown to be more prevalent in COVID-19 patients compared to non-COVID patients with nosocomial bacteremia, but with a similar proportion of *E. faecium* bacteremia (EfmB) [5].

Enterococcus species are known to have extensive antibiotic resistance patterns, including a broad range of both intrinsic and acquired resistance genes. They have a high number of mobile DNA elements, resulting from a frequent exchange of genetic material across species, genus, and family barriers [3]. Notably, enterococcal species demonstrate intrinsic resistance to many clinically important antibiotics, including non-penicillin beta-lactams, such as cephalosporins and most carbapenems, as well as to antibiotics that do not inhibit cell wall synthesis, such as aminoglycosides and clindamycin. While *E. faecalis* typically is sensitive to ampicillin and, subsequently, piperacillin, over 80% of *E. faecium* isolates in Sweden exhibit penicillin resistance [6], the corresponding figure being even higher in other regions [7]. Additionally, enterococci can acquire more resistance mechanisms, rendering them insensitive to vancomycin and teicoplanin, while resistance to linezolid and tigecycline remains rare [8]. Mechanisms of acquired antibiotic resistance in enterococci are complex and will not be addressed in this study.

Despite being rare in Sweden and other parts of Northern Europe [7], vancomycin-resistant enterococci (VRE) are of critical global concern. In February 2017, the WHO designated vancomycin-resistant *E. faecium* as one of the ESKAPE pathogens (an acronym for *Enterococcus faecium*, *Staphylococcus aureus*, *Klebsiella pneumoniae*, *Acinetobacter baumannii, Pseudomonas aeruginosa*, *Enterobacter* spp.), posing a significant threat to patients worldwide [9]. VRE was thereby given the highest priority status. VRE is still infrequent in most parts of Europe and extremely rare in Sweden, where vancomycin resistance is reported in less than 0.5 percent of enterococcal blood culture isolates [6,7].

Enterococci have traditionally been regarded as harmless commensal bacteria with low pathogenetic potential in healthy individuals. In spite of this, these organisms can give rise to severe infections, particularly in immunocompromised patients. Enterococci is today one of the most common causes of causes of gram-positive bacteremia in Europe and the United States [4,10]. Bacteremia with enterococci is mainly seen in fragile patients and is associated with a high rate of comorbidities. Especially for EfmB, conditions such as cancer, hematological malignancy, and neutropenia are frequently reported [11,12], while EfsB is associated with higher age, genitourinary focus, and cardiovascular risk factors, such as hypertension [13,14]. It is noteworthy that the incidence of EB has increased globally during the last decade [7], and EB is associated with high in-hospital mortality rates ranging from 11–36% [14,15,16]. *E. faecalis* is the more virulent species, but *E. faecium* is of increasing importance, as, in general, it is more resistant to antimicrobials [17]. Gudiol et al. showed that cancer patients with bacteremia caused by *E. faecium* more often received inadequate initial empirical antibiotic therapy than patients with bacteremia caused by *E. faecalis* [12]. Patients with EfmB also had a longer time to adequate empirical antibiotic therapy. Despite this, no significant differences were found between the two groups regarding outcomes such as early and overall mortality rates. However, there is a controversy in the literature regarding the association between *E. faecium* infection and mortality [13,16]. It is unclear whether any increase in mortality in EfmB patients is due to the infection itself or if the infection serves as a marker for underlying patient-related factors linked to nosocomial acquisition and underlying immunosuppression [18].

In Sweden, efforts to limit antibiotic resistance through stringent antibiotic policies favoring narrow-spectrum antibiotics have a longstanding tradition. Organizations behind the Swedish Strategic Program for the Rational Use of Antimicrobial Agents and Surveillance of Resistance (STRAMA) are well established, and their policies are integrated into everyday care also in the hospital sector. During the early 2000s, in the face of the emerging occurrence of extended-spectrum beta-lactamase (ESBL) in *Enterobactereales* and the first reported outbreak of multiresistant ESBL-producing *K. pneumoniae* in Scandinavia, a reduction in cephalosporin use was strongly favored by Strama and the Public Health Agency (Folkhälsomyndigheten) [19]. Consequently, there was an upsurge in the utilization of alternative antibiotics effective against Gram-negative or polymicrobial infections, such as piperacillin/tazobactam (pip/taz) [20]. However, this shift in antibiotic protocols may have inadvertently contributed to a selective pressure favoring ampicillin- and piperacillin-resistant enterococci, including *E. faecium*. The net effect of the selective pressure from the clinical use of different antibiotics is difficult to predict both at the individual patient level and on a broader scale. It involves several steps, including the disruption of the normal gut microbiome, which allows for an increase in enterococcal abundance in the gut flora. Additionally, sub-inhibitory effects of antibiotics not classified as valid treatment options in enterococcal infection, such as meropenem and quinolones, may serve as risk factors for the selection or development of beta-lactam-resistant enterococcal strains, as evident in the study by Gudiol [12].

The objective of this study was twofold: firstly, to evaluate whether the incidence of bacteremia with *E. faecium* relative to bacteremia with *E. faecalis* increased between 2015 and 2021 parallel to changing antibiotic prescription practices; secondly, to analyze risk factors associated with acquiring bacteremia with *E. faecium*, with particular focus on prior antibiotic exposure.

## 2. Materials and Methods

### 2.1. Study Population

This retrospective cohort study was conducted at Sahlgrenska University Hospital, a 1500-bed university hospital in Western Sweden. All patients over 18 years with positive blood cultures for *E. faecium* or *E. faecalis* between 2015 and 2021 were included in the patient population, while only patients with an EB in 2015, 2018, and 2021 were included in the medical record review and in the risk factor analyses. Patients were identified through a systematic search in the microbiological laboratory database. Each patient was included only once per year. Data on enterococcal species and antibiotic sensitivity were collected from the standard laboratory reports, and further microbiological analyses were not performed. Enterococcal species other than *E. faecium* or *E. faecalis*, patients with more than one enterococcal species, and those with restricted access to medical records were not included in the risk factor analyses. Patient data, including demographic and medical information, such as age, sex, the number of days in-hospital after positive blood culture, and if the bacteremia was hospital- or community-acquired, were collected in a standardized case report form through medical record review.

An episode of EB was defined as the presence of at least one positive blood culture containing either *E. faecium* or *E. faecalis*. The day of bacteremia onset was defined as the day of collection of the positive blood culture. Bacteremia was classified as hospital-acquired if the positive blood culture was obtained 48 h or more after hospital admission; otherwise, it was considered community-acquired. Other variables of interest included predisposing patient-related factors, such as comorbidities, prior hospital antibiotic exposure within 90 days preceding the positive blood culture, and the presence of drain ports, central lines, urinary catheters, or recent surgery. Outcome variables were mortality rates in-hospital and at 30 days, 90 days, and 1 year.

This study was conducted in accordance with the Declaration of Helsinki. Ethical approval was obtained from the ethics committee in Stockholm, Sweden, with approval number 2021-06683-01.

### 2.2. Statistical Analysis

Patients were categorized based on whether they had EfmB or EfsB. Continuous data were presented as median and interquartile ranges, while categorical variables were expressed as numbers and percentages. The statistical analysis was performed in SPSS statistics version 29, and a *p*-value below 0.05 was considered significant. Pearson Chi-square was used to compare categorical variables, and the Mann–Whitney U test was employed to compare medians between continuous data. Univariate and multivariate logistic regression models were used to identify risk factors for contracting EfmB over EfsB. Variables that exhibited statistical significance in the univariate analysis were incorporated into the multivariate regression analysis to ascertain their persistent significance after adjusting for potential confounding factors.

## 3. Results

### 3.1. Epidemiology and Demographics

In the years 2015, 2018, and 2021, 379 patients with enterococcal bacteremia were identified. After excluding patients with multiple species and those with restricted access to medical records, 171 patients with bacteremia caused by *E. faecium* and 189 with *E. faecalis* were included in the study (Table 1). In 2015, 98 unique patients had an episode of EB compared to 113 patients in 2020 and 149 patients in 2021, corresponding to a 52% crude increase in EB detection over seven years. Simultaneously, the total number of blood cultures processed at the Microbiological Laboratory at Sahlgrenska University Hospital increased from 46,573 in 2015 to 49,264 in 2018 and 52,790 in 2021, corresponding to a 13% increase.

**Figure 1:**
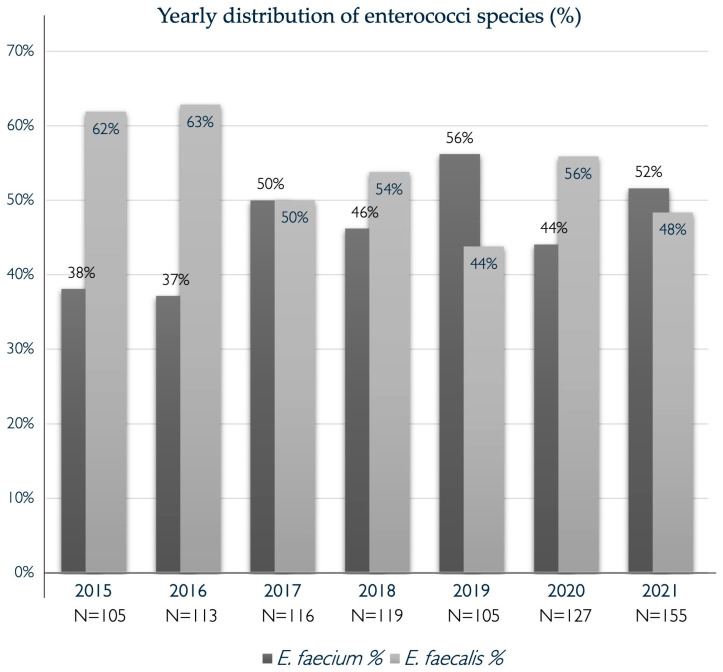
Occurrence and distribution of *Enterococci faecium* and *Enterococci faecalis* in blood cultures at Sahlgrenska University Hospital 2015–2021.

Altogether, 840 episodes of EB were included in the study population. The occurrence and distribution of bacteremia caused by *E. faecium* (*n* = 390, 46%*)* and *E. faecalis* (*n* = 450, 54%) were evaluated for the entire study period from 2015 to 2021. Although there was a rise in the number of EB cases over the years, this did not correspond to a statistically significant increase. Initially, the proportion of cases attributed to *E. faecium* was below 40%, with an upward trend in the later years of the study (Figure 1). All *E. faecalis* isolates and 9.9% of the *E. faecium* isolates were sensitive to ampicillin and pip/taz. No vancomycin-resistant enterococcal strains were detected during the entire study period. Additional sensitivity results for linezolid were reported in 2% of *E. faecalis* isolates and in 12% of *E. faecium* isolates, with all strains being sensitive to this antibiotic. Sensitivity testing for teicoplanin was performed in 3% of *E. faecium* isolates, all of which were sensitive. For tigecycline, sensitivity testing was performed in 10 *E. faecium* isolates, corresponding to 1% of the isolates, and not at all in *E. faecalis* isolates. One single isolate was reported as resistant to tigecycline but remained sensitive to vancomycin and linezolid. High- or low-grade aminoglycoside resistance was not regularly tested for.

Patients with EfsB were older (76 vs. 67 years; *p* < 0.001) compared to those with EfmB. The two enterococcal species were both more prevalent in males. Among comorbidities, hypertension was more prevalent in patients with EfsB (96/189, 51%) compared to those with EfmB (67/171, 39%) (*p* = 0.027). Hematological malignancy (28/179, 16% vs. 12/196, 6%, *p* = 0.003) and immunosuppression (48/171, 28% vs. 27/189, 14%, *p* = 0.002) were more common in patients with EfmB. Regarding predisposing procedures, the presence of a urinary catheter at the onset of bacteremia did not differ between the two sub-populations, while the use of a drain port, central vascular catheter, or recent surgery was more prevalent in patients with EfmB. Hospital acquisition was common in both groups but significantly more prevalent in EfmB (127/171, 74% vs. 69/189, 37%, *p* < 0.001). The hospital stay after the onset of bacteremia was longer in EfmB patients, 15 (10–31) vs. 10 (6–20) days, where *p* < 0.001. Bacteremia was monomicrobial in a majority of both EfmB and EfsB patients.

The unadjusted in-hospital mortality rates were 22% (42/189) and 20% (35/171) in the *E. faecalis* and *E. faecium* groups (ns). At 90 days post bacteremia onset, mortality reached 34% (65/189) in the *E. faecalis* group and 33% (56/171) in the *E. faecium* group. One-year mortality (assessed in 2015 and 2018) was 47% and 54%, respectively.

### 3.2. Changing Antibiotic Prescribing Practices

**Figure 2:**
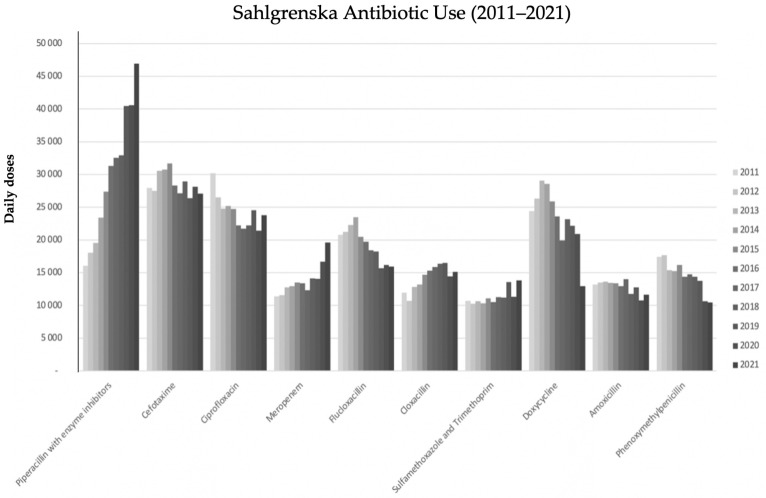
In-hospital antibiotic use 2011–2021 at Sahlgrenska University Hospital. Daily defined dose (DDD) according to the World Health Organization, except for cloxacillin, where a prescribed daily dose (PDD) of 6 g daily was applied.

From 2011 to 2021, the consumption of pip/taz increased from 16,000 to 47,000 daily defined doses (DDDs) per year at Sahlgrenska University Hospital (Figure 2). Notably, in 2016, pip/taz emerged as the most prescribed antibiotic in the hospital, with its prescription rate steadily escalating thereafter. This increase was partially offset by a reduction in the use of cephalosporins and ciprofloxacin, although not entirely compensated. Furthermore, there was a notable rise in meropenem usage over the years, with a particularly steep incline observed from 2020 to 2021, partly attributed to revised standard dose recommendations.

Data on antibiotic usage within 90 days before collection of the first positive blood culture with *E. faecalis* or *E. faecium* are presented in Table 2. Among patients with EfsB, 40 (21%) had received pip/taz, while the corresponding number was higher in patients with EfmB at 95 (56%; *p* < 0.001). The use of meropenem, the preferred carbapenem in the hospital, and ciprofloxacin, the predominant fluoroquinolone in use, was also more prevalent in patients with EfmB. A minority of the patients in both groups had not been prescribed any antibiotics within three months before the onset of bacteremia, and this was less common in the EfmB compared to the EfsB patients (9% vs. 38%; *p* < 0.001).

### 3.3. Variables Associated with E. faecium Bacteraemia

In the logistic regression analysis, several variables were found to be associated with an increased odds ratio (OR) of having bacteremia with *E. faecium* compared to *E. faecalis* (Table 3). Hospital acquisition exhibited an unadjusted OR of 5.02 (95% confidence interval (CI) 3.19–7.90) and an adjusted OR (aOR) of 2.23 (95% CI 1.19–4.15) for EfmB in comparison to EfsB. Other factors related to hospital care, such as prior surgery or the presence of a central venous catheter, urinary catheter, or surgical drain, demonstrated increased ORs for *E. faecium* in univariate comparison but not after adjusting for covariates.

If the patient had received pip/taz within 90 days before the date of the positive blood culture, the aOR for *E faecium* was 2.63 (95% CI 1.49–4.67) compared to experiencing bacteremia with *E. faecalis*. Similarly, if the patient had received meropenem, the aOR for EfmB was 4.26 with a 95% CI of 2.12–8.56. Moreover, the unadjusted OR for *E. faecium* associated with the use of ciprofloxacin was 2.44 (95% CI 1.46–4.10), but this was not significant after adjusting for other variables (an aOR of 1.88, 95% CI 0.98–3.63; *p* = 0.059).

## 4. Discussion

Our study, conducted over a seven-year period, 2015–2021, at Sahlgrenska University Hospital, aimed to investigate the potential increase in bacteremia caused by *E. faecium* in relation to changes in antibiotic use. While our findings did not establish a significant rise, a trend toward a higher proportion of *E. faecium* compared to *E. faecalis* in the later years of the study was observed. This trend aligns with similar observations documented internationally, indicating a global increase in the prevalence of *E. faecium* bacteremia [7,12,14,15,16]. Moreover, the annual number of EB appeared to increase over the study period. Similar patterns of rising prevalence of bacteremia caused by *E. faecium* have been documented internationally, including those in studies conducted in the United Kingdom, the Netherlands, Spain, Denmark, and the United States [12,21,22,23,24], although opposite trends have also been reported [13]. In contrast to the findings of rising EfmB incidence, a study from Switzerland reported an increase in *E. faecalis* cases [25]. Finally, compared to a Danish study from 2014, our cohort exhibited a higher proportion of *E. faecium* cases [16], while in another cohort study conducted over 10 years in Japan, *E. faecalis* accounted for 48% of cases, *E. faecium* for 30%, and other enterococcal species for 22% [26]. It is also worth stressing that vancomycin resistance in enterococci remains very uncommon in Sweden. This is demonstrated by the fact that none of the 840 individual enterococcal blood isolates in the present study exhibited resistance to vancomycin. This stands in stark contrast to the epidemiology in other parts of the world, where VRE accounts for over 40 percent of EB cases, as reported in the recent systematic review by Shiadeh et al. [7].

Notably, the reasons behind the shifts in enterococcal epidemiology remain complex and likely vary across different regions, time periods, and patient cohorts. However, the extensive in-hospital use of broad-spectrum antibiotics that promote intestinal colonization with enterococci, the intrinsic resistance of these bacteria to several commonly used antibiotics, and the capacity of enterococcal strains to acquire and disseminate antibiotic resistance determinants have been suggested as driving factors [27].

Additionally, our study identified several important demographic and clinical factors that differentiate patients with bacteremia caused by *E. faecium* from those with *E. faecalis.* Patients with EfsB were older and exhibited a higher prevalence of hypertension, while hematological malignancy and immunosuppression were more common in the EfmB patients. This is in line with a study of EB in cancer patients, where *E. faecium* tended to be more prevalent in patients with hematological malignancies compared to other malignancies [12] and was also documented in two epidemiological studies [13,16]. From their findings, Billington et al. argued that *E. faecalis* and *E. faecium* should be regarded as two clinically different entities with unique sets of risk factors and microbiologic characteristics. Additionally, certain predisposing procedures, including the presence of a central vascular catheter or recent surgery, were more commonly observed in patients with EfmB. The relatively high frequency of *E. faecium* in our cohort can probably be attributed to the tertiary care setting at Sahlgrenska University Hospital, which includes facilities for solid organ transplantation in adults and pediatric patients as well as advanced hematological units with stem cell transplantation and specialized oncology departments, among other advanced medical facilities. These units treat highly vulnerable and immunodeficient patients prone to nosocomial infections. As expected, there was an independently higher OR for the nosocomial origin of bacteremia caused by *E. faecium* compared to *E. faecalis,* albeit nosocomial acquisition was also frequent in EfsB patients.

Mortality rates, both in hospital and during one-year follow-up, were similar in patients with bacteremia caused by *E. faecium* and *E. faecalis*, at around 20% and 50%, respectively. This was seen even though EfsB patients were a median of 9 years older when contracting their bacteremia. On the other hand, a significant proportion of EfmB patients were immunocompromised and had underlying hematological malignancies. This finding aligns with the broader discourse concerning the relationship between *E. faecium* infection and mortality. The extent to which mortality in EfmB patients is directly attributable to the infection itself remains unclear, and results from previous studies are contradictory [12,13,14,16,28]. It is possible that the occurrence of EfmB serves more as an indicator of underlying patient-related factors, such as conditions associated with nosocomial acquisition and immunosuppression, rather than being a primary cause of death.

Additionally, EfmB patients more often had been treated with meropenem or pip/taz prior to their episode of EB and more frequently had a nosocomial infection. Interestingly, in our population, the trend toward a higher proportion of EfmB coincided with increased use of the antibiotic pip/taz, which, from 2016, was the most commonly used antibiotic in the hospital. Pip/taz has an excellent antimicrobial effect against *E. faecalis,* while 90% of *E. faecium* strains in the study were resistant. Pip/taz is also effective against most anaerobic bacteria dominating the distal intestinal flora. Consequently, the shift in antibiotic prescription practices in Sweden favoring increased empirical use of pip/taz instead of cephalosporines, advocated for by the stewardship organizations in the mid-2000s to meet the surge of ESBL-producing *Enterobacterales*, probably has contributed to a higher colonization density with *E. faecium* in the individual patient. A shift toward more contagious nosocomial clones of *E. faecium* within the hospital flora might have also occurred [4]. The substantial increase in the utilization of pip/taz over the study period, along with a concurrent rise in meropenem usage, reflects changes in treatment preferences and antimicrobial stewardship practices. These modifications aimed to address shifts in antimicrobial resistance in pathogenic bacteria and meet the medical needs of vulnerable patients. However, they might also have led to unforeseen consequences [15]. The extent to which the adoption of pip/taz as the most prescribed antibiotic in the hospital has contributed to the observed trends in EB epidemiology remains unclear. Alternative broad-spectrum antibiotics that may be used also exert selective pressure on microbial populations. Therefore, further surveillance and investigations are warranted to elucidate the complex interplay between antibiotic utilization patterns and the emergence of antimicrobial resistance. Antibiotic exposure within 90 days prior to the onset of bloodstream infection was very common, observed in 91% of patients with EfmB and 62% of patients with EfsB. Notably, exposure to pip/taz was independently associated with a higher risk of *E. faecium*, with an adjusted odds ratio of 2.63 in the logistic regression model. The relationship between previous meropenem exposure and increased odds for *E. faecium* was even stronger, which was somewhat unexpected but consistent with other studies [12]. The findings suggest that meropenem, particularly at high concentrations, may have an effect on ampicillin-susceptible enterococci, even though this antibiotic is generally considered ineffective against *E. faecalis* [29]. The sequential use of both antibiotics in individual patients and other unaccounted patient-related factors may also contribute to this association. Although there was an association between previous ciprofloxacin use and subsequent EfmB in the univariate comparison, it did not remain significant after adjustment. However, a selective pressure of fluoroquinolone on enterococci, regardless of species, is likely.

Our study has several limitations that should be considered when interpreting the results. Firstly, it is a retrospective study, which inherently comes with certain limitations, including reliance on existing medical records and the potential for bias in data collection. Furthermore, the COVID-19 pandemic, particularly the second wave, which occurred during the final years of the study, may have influenced our findings, which has been documented in other studies [5]. The pandemic, apart from stressing the entire healthcare system, likely led to changes in healthcare-seeking behavior, hospital admissions, and antibiotic prescribing practices, which could have impacted the incidence and characteristics of EB cases included in our study. Moreover, there are inherent differences between patients prone to bacteremia caused by *E. faecium* and those prone to *E. faecalis,* including underlying comorbidities, immune status, and healthcare exposures, among others. Additionally, the study was conducted at a single tertiary care center, which may limit the generalizability of our findings to other healthcare settings or populations. Despite these limitations, the present study provides valuable insights into the epidemiology and clinical characteristics of enterococcal bacteremia in a VRE low-incidence area, contributing to the existing body of literature on this topic. Future research, including prospective studies and multi-center collaborations, is warranted to further elucidate risk factors influencing the incidence and outcomes of EB and to inform evidence-based interventions for its prevention and management.

## 5. Conclusions

In conclusion, our study reveals that bacteremia caused by *E. faecalis* and *E. faecium* largely afflict different patient populations. We also observed an upward trend in both the number and proportion of EfmB cases during the study period from 2015 to 2021. Notably, our analysis identified three independent variables associated with a higher likelihood of acquiring *E. faecium*. These include nosocomial infection and prior exposure to either pip/taz or meropenem within 90 days before the bacteremia episode. Although the reasons behind the rise in EfmB incidence remain unclear, one potential contributing factor could be changes in antibiotic usage patterns within the hospital during this period.

## Figures and Tables

**Table 1:** Demographic and clinical characteristics of study patients 2015, 2018, and 2021.

	*E. faecalis n* = 189 (%)	*E. faecium n* = 171 (%)	*p*
2015 *n* = 98	58/98 (59)	40/98 (41)	ns
2018 *n* = 113	59/113 (52)	54/113 (48)	ns
2021 *n* = 149	72/149 (49)	77/149 (51)	ns
*Demographics*			
Age (years)	76 (67–83)	67 (56–75)	<0.001
Women	56 (30)	66 (39)	ns
In-hospital stay (days)	36 (30–54)	68 (56–90)	<0.001
Bacteremia duration ^1^	10 (6–20)	15 (10–31)	<0.001
Hospital-acquired bacteremia	69 (37)	127 (74)	<0.001
*Comorbidities*			
Diabetes	48 (25)	37 (22)	ns
Chronic kidney disease	31 (16)	20 (14)	ns
Hypertension	96 (51)	67 (39)	0.027
Heart failure	27 (14)	16 (9)	ns
Colon cancer	13 (7)	15 (9)	ns
Hematological malignancy	12 (6)	28 (16)	0.003
Other cancer	55 (29)	49 (29)	ns
COPD ^2^	15 (8)	13 (8)	ns
Liver failure	21 (11)	19 (11)	ns
Gastric ulcer	14 (7)	18 (11)	ns
IBD ^3^	3 (2)	4 (2)	ns
Immunosuppression	27 (14)	48 (28)	0.001
Dementia	13 (7)	7 (4)	ns
No comorbidities	11 (6)	10 (6)	ns
*Predisposing hospital procedures*			
Urine catheter	93 (49)	94 (55)	ns
Drain port	30 (16)	58 (34)	<0.001
Central vascular catheter	55 (29)	116 (68)	<0.001
Recent surgery	73 (39)	101 (59)	<0.001
*Mortality*			
In-hospital	42 (22)	35 (20)	ns
30 days	51 (27)	41 (24)	ns
90 days	65 (34)	56 (33)	ns
1 year ^4^	54/116 (47)	49/94 (54)	ns

Continuous data are presented as median and interquartile range. Categorical variables are listed as numbers and percentages. ^1^ From bacteremia onset to hospital discharge. ^2^ Chronic obstructive pulmonary disease. ^3^ Inflammatory bowel disease. ^4^ Assessed in 2015 and 2018. ns = not significant.

**Table 2:** Antibiotic treatment within 90 days before positive blood culture.

	*E. faecalis* *n* = 189 (%)	*E. faecium* *n* = 171 (%)	*p*
Pip/taz ^1^	40 (21)	95 (56)	<0.001
Cephalosporins	32 (17)	42 (22)	ns
Meropenem	14 (7)	71 (42)	<0.001
Ciprofloxacin	28 (15)	51 (30)	<0.001
No antibiotics	71 (38)	15 (9)	<0.001

Variables are listed as numbers and percentages. ^1^ Piperacillin/tazobactam. ns = not significant.

**Table 3:** Univariate and multivariate logistic regression model for variables associated with *Enterococcus faecium* bacteremia.

	OR ^1^ (95% CI ^2^)	*p*	aOR ^3^ (95% CI ^2^)	*p*
Age	0.96 (0.95–0.98)	<0.001		
Hospital-acquired	5.02 (3.19–7.90)	<0.001	2.23 (1.19–4.15)	0.012
Hypertension	0.62 (0.41–0.95)	0.027		
Hematological malignancy	2.89 (1.42–5.88)	0.003		
Immunosuppression	2.34 (1.38–3.96)	0.002		
Drain port	2.72 (1.65–4.50)	<0.001		
Central vascular catheter	5.14 (3.28–8.05)	<0.001		
Recent surgery	2.29 (1.50–3.50)	<0.001		
Prior antibiotic use within 90 days				
Pip/taz ^4^	4.66 (2.94–7.39)	<0.001	2.63 (1.49–4.67)	<0.001
Carbapenems	8.88 (4.76–16.56)	<0.001	4.26 (2.12–8.56)	<0.001
Quinolones	2.44 (1.46–4.10)	<0.001		
No antibiotics	0.16 (0.09–0.29)	<0.001		

Only statistically significant variables are displayed in the multivariate analysis column. ^1^ Odds ratio. ^2^ Confidence interval. ^3^ Adjusted odds ratio. ^4^ Piperacillin/tazobactam.

## Data Availability

The original contributions presented in the study are included in the article. Further inquiries can be directed to the corresponding authors.

## References

[1] Ramos S., Silva V., Dapkevicius M.L.E., Igrejas G., Poeta P. (2020). Enterococci, from Harmless Bacteria to a Pathogen. Microorganisms.

[2] Schleifer K.H., Kilpper-Bälz R. (1984). Transfer of *Streptococcus faecalis* and *Streptococcus faecium* to the Genus Enterococcus nom. rev. as *Enterococcus faecalis* comb. nov. and *Enterococcus faecium* comb. nov. Int. J. Syst. Evol. Microbiol..

[3] García-Solache M., Rice L.B. (2019). The Enterococcus: A Model of Adaptability to Its Environment. Clin. Microbiol. Rev..

[4] Arias C.A., Murray B.E. (2012). The rise of the *Enterococcus*: Beyond vancomycin resistance. Nat. Rev. Microbiol..

[5] Buetti N., Tabah A., Loiodice A., Ruckly S., Aslan A.T., Montrucchio G., Cortegiani A., Saltoglu N., Kayaaslan B., Aksoy F. (2022). Different epidemiology of bloodstream infections in COVID-19 compared to non-COVID-19 critically ill patients: A descriptive analysis of the Eurobact II study. Crit. Care.

[6] Folkhälsomyndigheten (2022). Swedres-Svarm 2022. https://www.sva.se/media/ticcp2zu/swedres-svarm-2022-edit-230808.pdf.

[7] Jabbari Shiadeh S.M., Pormohammad A., Hashemi A., Lak P. (2019). Global prevalence of antibiotic resistance in blood-isolated *Enterococcus faecalis* and *Enterococcus faecium*: A systematic review and meta-analysis. Infect. Drug Resist..

[8] Kristich C.J.R.L., Arias C.A., Gilmore M.S., Clewell D.B., Ike Y., Shankar N. (2014). Enterococcal Infection—Treatment and Antibiotic Resistance. Enterococci: From Commensals to Leading Causes of Drug Resistant Infection.

[9] World Health Organization (2017). Prioritization of Pathogens to Guide Discovery, Research and Development of New Antibiotics for Drug-Resistant Bacterial Infections, Including Tuberculosis.

[10] Pérez-Crespo P.M.M., Lanz-García J.F., Bravo-Ferrer J., Cantón-Bulnes M.L., Domínguez A.S., Aguirre J.G., Reguera-Iglesias J.M., Jiménez E.L., Castillo C.A., Vallejo M.Á.M. (2021). Revisiting the epidemiology of bloodstream infections and healthcare-associated episodes: Results from a multicentre prospective cohort in Spain (PRO-BAC Study). Int. J. Antimicrob. Agents.

[11] Suppola J.P., Kuikka A., Vaara M., Valtonen V.V. (1998). Comparison of risk factors and outcome in patients with Enterococcus faecalis vs Enterococcus faecium bacteraemia. Scand. J. Infect. Dis..

[12] Gudiol C., Ayats J., Camoez M., Domínguez M.Á., García-Vidal C., Bodro M., Ardanuy C., Obed M., Arnan M., Antonio M. (2013). Increase in bloodstream infection due to vancomycin-susceptible Enterococcus faecium in cancer patients: Risk factors, molecular epidemiology and outcomes. PLoS ONE.

[13] Billington E.O., Phang S.H., Gregson D.B., Pitout J.D.D., Ross T., Church D.L., Laupland K.B., Parkins M.D. (2014). Incidence, risk factors, and outcomes for *Enterococcus* spp. blood stream infections: A population-based study. Int. J. Infect. Dis..

[14] McBride S.J., Upton A., Roberts S.A. (2010). Clinical characteristics and outcomes of patients with vancomycin-susceptible *Enterococcus faecalis* and *Enterococcus faecium* bacteraemia—A five-year retrospective review. Eur. J. Clin. Microbiol. Infect. Dis..

[15] Cabiltes I., Coghill S., Bowe S.J., Athan E. (2020). Enterococcal bacteraemia ‘silent but deadly’: A population-based cohort study. Intern. Med. J..

[16] Pinholt M., Østergaard C., Arpi M., Bruun N.E., Schønheyder H.C., Gradel K.O., Søgaard M., Knudsen J.D., Danish Collaborative Bacteraemia Network (DACOBAN) (2014). Incidence, clinical characteristics and 30-day mortality of enterococcal bacteraemia in Denmark 2006–2009: A population-based cohort study. Clin. Microbiol. Infect..

[17] Karaman R., Jubeh B., Breijyeh Z. (2020). Resistance of Gram-Positive Bacteria to Current Antibacterial Agents and Overcoming Approaches. Molecules.

[18] Echeverria-Esnal D., Sorli L., Navarrete-Rouco M.E., Prim N., Barcelo-Vidal J., Conde-Estévez D., Montero M.M., Martin-Ontiyuelo C., Horcajada J.P., Grau S. (2023). Ampicillin-resistant and vancomycin-susceptible *Enterococcus faecium* bacteremia: A clinical narrative review. Expert Rev. Anti Infect. Ther..

[19] Lytsy B., Sandegren L., Tano E., Torell E., Andersson D.I., Melhus A. (2008). The first major extended-spectrum beta-lactamase outbreak in Scandinavia was caused by clonal spread of a multiresistant *Klebsiella pneumoniae* producing CTX-M-15. Apmis.

[20] STRAMA (2022). 10-Punktsprogram Mot Antibiotikaresistens Inom Vård och Omsorg. https://strama.se/wp-content/uploads/2022/06/10-punktsprogrammet-uppdaterad-kort-version-juni-2022.pdf.

[21] Horner C., Mushtaq S., Allen M., Hope R., Gerver S., Longshaw C., Reynolds R., Woodford N., Livermore D.M. (2021). Replacement of *Enterococcus faecalis* by *Enterococcus faecium* as the predominant enterococcus in UK bacteraemias. JAC Antimicrob. Resist..

[22] Lester C.H., Sandvang D., Olsen S.S., Schønheyder H.C., Jarløv J.O., Bangsborg J., Hansen D.S., Jensen T.G., Frimodt-Møller N., Hammerum A.M. (2008). Emergence of ampicillin-resistant *Enterococcus faecium* in Danish hospitals. J. Antimicrob. Chemother..

[23] Top J., Willems R., Blok H., De Regt M., Jalink K., Troelstra A., Goorhuis B., Bonten M. (2007). Ecological replacement of *Enterococcus faecalis* by multiresistant clonal complex 17 *Enterococcus faecium*. Clin. Microbiol. Infect..

[24] Top J., Willems R., Bonten M. (2008). Emergence of CC17 *Enterococcus faecium*: From commensal to hospital-adapted pathogen. FEMS Immunol. Med. Microbiol..

[25] Piezzi V., Gasser M., Atkinson A., Kronenberg A., Vuichard-Gysin D., Harbarth S., Marschall J., Buetti N. (2020). Increasing proportion of vancomycin-resistance among enterococcal bacteraemias in Switzerland: A 6-year nation-wide surveillance, 2013 to 2018. Eurosurveillance.

[26] Suzuki H., Hase R., Otsuka Y., Hosokawa N. (2017). A 10-year profile of enterococcal bloodstream infections at a tertiary-care hospital in Japan. J. Infect. Chemother..

[27] Mancuso G., Midiri A., Gerace E., Biondo C. (2021). Bacterial Antibiotic Resistance: The Most Critical Pathogens. Pathogens.

[28] Lupia T., Roberto G., Scaglione L., Shbaklo N., De Benedetto I., Scabini S., Mornese Pinna S., Curtoni A., Cavallo R., De Rosa F.G. (2022). Clinical and microbiological characteristics of bloodstream infections caused by *Enterococcus* spp. within internal medicine wards: A two-year single-centre experience. Intern. Emerg. Med..

[29] Endtz H.P., van Dijk W.C., Verbrugh H.A. (1997). Comparative in-vitro activity of meropenem against selected pathogens from hospitalized patients in The Netherlands. MASTIN Study Group. J. Antimicrob. Chemother..

